# Interventions Aimed at Increasing Dairy and/or Calcium Consumption of Preschool-Aged Children: A Systematic Literature Review

**DOI:** 10.3390/nu11040714

**Published:** 2019-03-27

**Authors:** Victoria Srbely, Imtisal Janjua, Andrea C. Buchholz, Genevieve Newton

**Affiliations:** 1 Department of Human Health & Nutritional Science, University of Guelph, Guelph, ON N1G2W1, Canada; vsrbely@uoguelph.ca; 2 Department of Biomedical Sciences, University of Guelph, Guelph, ON N1G2W1, Canada; ijanjua@uoguelph.ca; 3 Department of Family Relations and Applied Nutrition, University of Guelph, Guelph, ON N1G2W1, Canada; abuchhol@uoguelph.ca

**Keywords:** intervention, nutrition, preschool, child, parent, dairy, calcium

## Abstract

Dairy product consumption is important during childhood, as dairy products provide nutrients to support growth and development. However, a high proportion of children globally are not meeting recommended daily intakes, which may have long-term health implications. Accumulating evidence suggests that interventions aimed at instilling healthy lifestyle habits are most effective when initiated during the preschool years. Therefore, the purpose of the review was to identify the characteristics of effective dairy and/or calcium interventions targeting preschool-aged children. A systematic literature review identified 14 intervention studies published between 1998–2018 addressing dairy/calcium intakes in the preschool population (1.5 to 5 years). Intervention reporting was assessed using intervention intensity, behavior change techniques and Workgroup for Intervention Development and Evaluation Research (WIDER), with the quality of studies evaluated using risk of bias and Grades of Recommendation, Assessment, Development and Evaluation (GRADE). Five of the 14 studies included in the review reported significant improvements in children’s dairy (4/5) or calcium (1/5) intake. Characteristics that may enable intervention effectiveness include the delivery of interventions in one setting (preschool facility), using specific behavior change techniques (environmental restructuring and teach to use prompts/cues), and targeting both parent and child. Overall, the interventions assessed demonstrated variable success and highlighted the need for developing effective interventions designed to increase dairy and/or calcium intakes in preschool-aged children.

## 1. Introduction

Consumption of dairy products is an important determinant of childhood health and development [1]. Dairy products such as milk, yogurt, and cheese have a rich nutrient profile which includes both macronutrients and micro-nutrients (calcium, vitamin D) that support the optimization and maintenance of good health [2]. Despite the importance of dairy consumption, studies have demonstrated that a significant number of North American children are not meeting recommended intakes, as based on the 2018 Canadian Food Guide [3]. In Canada, 37% of children aged four to nine years do not consume the recommended number of servings of milk and alternatives [3]. Furthermore, between 1977–2001, the proportion of children aged two to 18 years in the United States (U.S.) consuming milk decreased from 94% to 84%, the number of servings consumed decreased from 3.5 to 2.8/day, and the portion size of each serving decreased from 460 to 410 mL [4]. Similar findings have been reported in Europe. For example, the Individuelle Nationale sur les Consommations Alimentaires study conducted in France between 1999–2007 reported a decrease in milk and cheese consumption of 10% and 12%, respectively, in children aged 3 to 10 years old [5]. The Dortmund Nutritional and Anthropometric Longitudinally Designed Study (1986–2001) comprised of German children between the ages of 1–13 years old further demonstrated the global decline in dairy consumption by also reporting a negative trend in milk consumption (−6.5 g/day/study year for children aged 1 to 3; −2.8 to −7.4 g/day/study year for children aged 4 to 13) [5]. A decline in dairy intake from early childhood may have important implications on bone health [6], as well as on the risk of developing a number of costly chronic health conditions such as obesity [7,8], type 2 diabetes [9,10], hypertension [9], and colorectal cancer [11].

Accumulating evidence strongly suggests that interventions aiming to instill healthy lifestyle habits and prevent chronic diseases are most effective when initiated during preschool (≤5 y), as compared to school-age (>5 y) years [12]. For example, Skinner et al. found that the consumption of fruits among school-aged children was predicted by exposure to and consumption of a variety of fruits during preschool years [13]. Marshall et al. (2005) similarly reported that the intake of carbonated beverages, juice drinks, and sugar-sweetened beverages was inversely associated with milk intake in a study of 645 children aged 1 to 5, suggesting the displacement of milk by other beverages [14]. This displacement has potentially significant health consequences, as reported by Dubois et al. (2007), who found that 15.4% of preschoolers in the Québec Longitudinal Study of Child Development who were regular consumers of sugar-sweetened beverages were overweight at 4.5 years of age, as compared to only 6.9% of non-consumers [15]. Furthermore, U.S. data suggest that for each 30-mL reduction in milk consumption by children aged five to 18 years, sugar-sweetened beverage consumption increases by 126 mL, resulting in a net increase of 31 kcal and a loss of 34 mg of calcium for each 30 mL of milk displaced [16]. Collectively, these findings reinforce the need to investigate factors influencing children’s dairy intakes during preschool years. Previously published systematic literature reviews have investigated the effects of dietary interventions aiming to increase dairy consumption; however, they targeted children aged 5 to 12 [17] and adolescents aged ≥12 to ≤18 [18], so there is a gap in knowledge regarding dairy intakes in the preschool population.

Enhancing dairy and calcium intake in preschool children is potentially an important step in optimizing children’s bone health [6] and mitigating other long-term health consequences associated with insufficient intake. Therefore, the objective of this systematic literature review was to identify the characteristics that constitute effective dairy and/or calcium interventions targeting preschool-aged children. The results of this review will inform the development of future dairy/calcium intervention studies and public health education efforts.

## 2. Materials and Methods

### 2.1. Search Method

A list of search terms and keywords were adopted from recent and relevant reviews [17,18], and modified with assistance from an open education resources librarian. The search terms were comprehensive and inclusive, highlighting dairy and/or calcium consumption in preschool-aged children. The search terms were categorized under four headings:

1. Interventions (e.g., intervention, clinical trial, experimental studies)
2. Nutrition (e.g., diet, food, beverage)
3. Population (e.g., preschool, toddler and parent, family)
4. Dairy/Calcium (e.g., yogurt, dairy, milk).

The databases searched included ProQuest, Web of Science, Cochrane Database, Cumulative Index to Nursing and Allied Health Literature, CAB Direct, and PsycINFO. Varying combinations of search terms and keywords were applied to each of the six databases searched (see Supplementary Materials). This allowed for Medical Subject Headings (MeSH) terms to be used along with keywords, where permitted. The final literature search was conducted on 14 June 2018. It included all papers published between 1998–2018, and was restricted to English publications. Grey literature and reference lists of review papers were searched for additional intervention studies focused on dairy consumption. All of the study protocols were registered on 17 July 2018 in PROSPERO (international database of prospectively registered systematic reviews) under the study identification code CRD42018099909.

### 2.2. Inclusion Criteria

Inclusion criteria limited the selection of studies to: (i) intervention studies with or without control groups, (ii) intervention studies modifying dietary intakes (specifically including dairy and/or calcium intake as a measurement), (iii) the primary or secondary aim of the study being an increase in dairy and/or calcium consumption in preschool-aged children (1.5 to 5 years), including any studies that targeted families (parents and/or children), schools, and/or early education centers, and (iv) intervention studies reporting changes in dairy and/or calcium intakes at either the individual and/or group level.

### 2.3. Exclusion Criteria

Exclusion criteria included: (i) studies targeting clinical populations (i.e., obese or lactose intolerant groups), (ii) case studies, (iii) studies focused on breast-feeding, allergies, or calcium supplementation, (iv) studies aimed at changing the type of dairy consumption (i.e., regular-fat dairy to low-fat dairy, but not total dairy), (v) publications older than 20 years (i.e., published prior to 1998), and (vi) non-English publications.

### 2.4. Data Extraction and Synthesis

**Figure 1:**
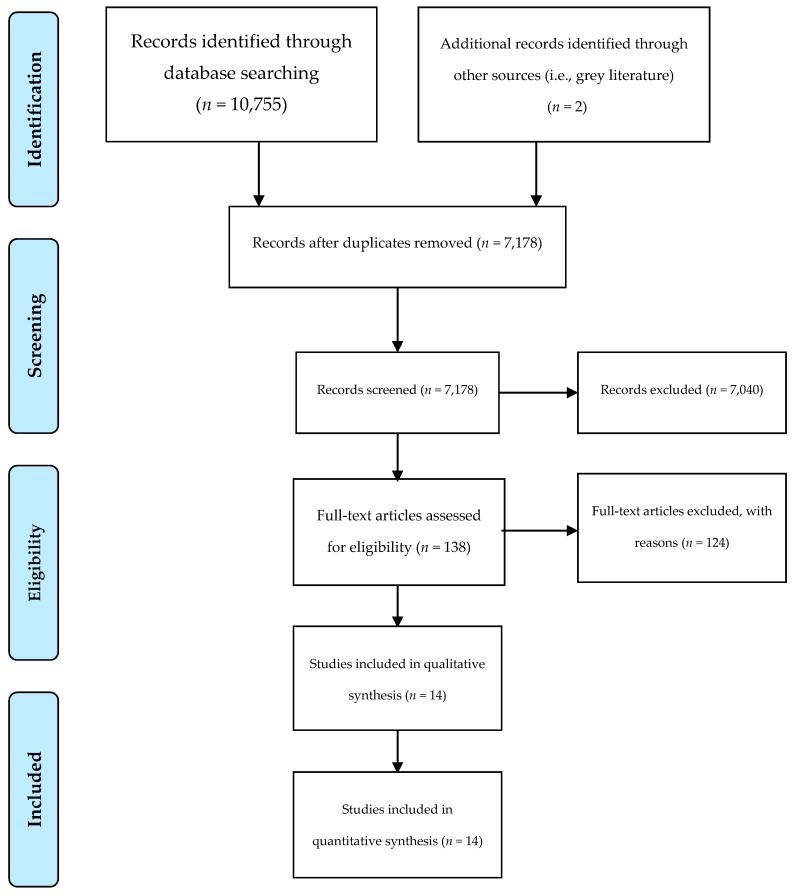
Preferred Reporting Items for Systematic Reviews and Meta-Analyses (PRISMA) four-phase flow diagram of the literature search results. From: Moher, D.; Liberati, A.; Tetzlaff, J.; Altman, D.G.; The PRISMA Group. Preferred reporting items for systematic reviews and meta-analyses: The PRISMA statement. *PLoS Med.* **2009**, *6*, e1000097, doi:10.1371/journal.pmed1000097 [21]. For more information, visit www.prisma-statement.org.

The Preferred Reporting Items for Systematic Reviews and Meta-Analyses (PRISMA) diagram summarizing the search outcomes is presented in Figure 1. A total of 7178 records were screened (7176 records identified through database searching, two records [19,20] identified through grey literature searching), with 138 articles assessed in full-text based on the specified inclusion and exclusion criteria. A total of 124 articles were excluded based on a lack of access to full-text English publication (*n* = 18), incorrect age range (*n* = 38), not aiming to increase the consumption of dairy and/or calcium (*n* = 23), aiming to change the type of dairy consumption (*n* = 16), focusing on dairy and/or calcium supplementation (*n* = 6), being a case study (*n* = 1), or no intervention (*n* = 22). A total of 14 intervention studies were included in the final analysis.

Two independent reviewers (VS, IJ) extracted data from the studies, including the author name and date, population characteristics, description of the intervention, relevant outcome measures, effect size, and effectiveness of the intervention (Table 1). Five assessment tools, including (1) Intervention Intensity Analysis, (2) Coventry, Aberdeen, and London—Refined Taxonomy of Behavior Change Techniques, (3) Workgroup for Intervention Development and Evaluation Research Recommendations, (4) Cochrane Collaboration Risk of Bias Tool, and (5) Grades of Recommendation, Assessment, Development and Evaluation were used to evaluate all of the intervention studies included in the analysis. The five assessment tools are described below.

### 2.5. Intervention Reporting

#### 2.5.1. Intervention Intensity

Intervention intensity scales have been employed in scientific literature to enhance comparisons of interventions between studies [17]. An intervention intensity scale is a point-scale assessment tool that evaluates the characteristics and degree of an intervention [17]. The intensity score ranks the qualities of each individual intervention as high, medium, or low intensity, facilitating straightforward comparisons of different study designs and/or intervention settings [17].

The intervention intensity scale used was adapted from a recent review [18], assessing four characteristics of interventions on a 5-point ranking scale (1 = low, 2 = low-medium, 3 = medium, 4 = medium–high, and 5 = high), with the exception of “reach of the intervention strategies” [34]. The four characteristics are detailed below:

(1) *Duration of the intervention.* This category ranked the length of the intervention using the following scale: 1 = ≤6 weeks, 2 = 6 to 11 weeks, 3 = 12 weeks to 5 months, 4 = 6 to 12 months, and 5 = ≥12 months.
(2) *Frequency of contact with the intervention.* This characteristic assessed the frequency of contact between participants and the intervention. If the intervention employed multiple points of contact, an average contact score was computed. If the frequency of contact was not clearly stated by authors, the points of contact were divided by the overall duration of the intervention to determine an average frequency of contact. The ranking score that was used for frequency of contact with the intervention was 1 = annually, 2 = bimonthly to quarterly, 3 = monthly, 3.5 = twice a month, 4 = weekly, 4.5 = multiple times per week, and 5 = daily.
(3) *Level of personalization.* This characteristic describes the type and/or level of contact with the intervention. The ranking score used for the level of personalization included: 1 = environmental, 2 = group (parent or child), 2.5 = group (parent and child), 3 = environmental and group (parent or child), 3.5 = environmental and group (parent and child), 4 = group with an individual component (parent or child), 4.5 = group with an individual component (parent and child), and 5 = individual (parent and/or child) or individual, environmental, and group (parent and/or child). If the parent and child experienced different levels of personalization, they were scored independently, and the scores were averaged for a total personalization score out of 5. The more personalized the contact of the intervention, the higher the intensity score.
(4) *Reach of the intervention strategies*. This characteristic assessed the number of different settings (i.e., home, school) used by the researchers to reach their target audience, and used a scale where 1 = one setting, 3 = two settings, and 5 = three or more settings. The greater number of settings used with the intervention, the higher the intensity of the intervention.

The two reviewers (VS, IJ) scored the characteristics and provided an overall intervention intensity score for each intervention included in the analysis. The overall intensity score was the sum of the scores of the four characteristics, giving a total score out of 20. An overall intervention intensity score of greater than or equal to 13.5 was considered a high-intensity intervention, between 10.51–13.49 was rated as medium intensity, and a score of 10.5 or less indicated a low-intensity intervention.

#### 2.5.2. Behavior Change Techniques

Michie et al. (2011) published the Coventry, Aberdeen, and London-Refined (CALO-RE) Taxonomy of Behavior Change Techniques to be used in assessment of interventions targeting healthy eating and physical activity [35]. The refined taxonomy published by Michie et al. (2011) was adopted from the taxonomy of theory-linked behavior change techniques developed by Abraham and Michie (2008) [36], which identified specific behavior change techniques in interventions that enabled effectiveness. The twofold rationale for the use of the CALO-RE Taxonomy is based on determining whether the differences in behavior change techniques observed across studies impacted the effectiveness of each intervention, and secondly, identifying which techniques affected the most significant behavioral change [36].

The CALO-RE Taxonomy provides a behavior change taxonomy of 40 items, which are defined in Supplementary Materials Table S1. Two independent reviewers (VS, IJ) evaluated the behavior change techniques applied in the intervention studies and resolved any discrepancies through discussion. Behavior change techniques three, six, 11, 12, 14, 17, 18, 31, 32, 33, 34, 37, and 40 were excluded from analysis, as these techniques were not employed in any of the interventions assessed.

#### 2.5.3. WIDER Recommendations

The Workgroup for Intervention Development and Evaluation Research (WIDER) [37] developed a framework to assess and report the components of behavior change intervention studies, recommending a set of four criteria by which to evaluate techniques employed in behavior change interventions. The WIDER recommendations were developed to compare behavior change interventions across heterogeneous studies, with the goal of ensuring clarity in reporting of the components of behavior change techniques to ultimately improve the reproducibility of current intervention methods. The description of the four criteria of the WIDER recommendations are outlined in Supplementary Materials Table S2 and detailed below:

(1) The first recommendation addresses the description of the intervention(s) and the level of detail reported by authors. There are eight supplementary recommendations required for discussion throughout the intervention study, including the characteristics of those delivering the intervention, characteristics of the recipients, setting, mode of intervention delivery, intensity, duration, adherence to delivery protocols, and a detailed description of the intervention content for each study group.
(2) The second recommendation addresses the change process employed in the intervention and the design of the intervention. This recommendation requires a description of how the intervention was developed, the behavior change techniques used in the intervention, and the behavioral processes being targeted by the change techniques.
(3) The third recommendation addresses the extent to which the intervention protocols and/or manuals are accessible, as authors must provide easy access to the protocols/manuals for the interventions as supplementary materials (i.e., online).
(4) The fourth recommendation assesses the control group and the control conditions. Authors must describe the characteristics of the interveners delivering the control, characteristics of the control participants, setting, mode of delivery, intensity, duration, compliance to the delivery protocols, and a detailed description of the control content.

Two reviewers (VS, IJ) independently assessed all of the intervention studies using the four WIDER recommendations, and reported whether each intervention satisfied all of the subcomponents of the recommendations [38].

### 2.6. Quality Criteria

#### 2.6.1. Risk of Bias

The Cochrane Risk of Bias Tool [39] was used to evaluate six types of bias in the individual studies, including selection bias, performance bias, attrition bias, reporting bias, detection bias, and other bias. Within the six types of bias, seven domains exist that aid in assessing the risk of each type of bias:

(1) *Selection bias*: assessed two domains: sequence generation and allocation concealment
(2) *Performance bias*: assessed the blinding procedures implemented in the study
(3) *Detection bias*: assessed the adequacy of the blinding of outcome assessors
(4) *Attrition bias*: assessed all the participant withdrawal from the study that lead to incomplete outcome data
(5) *Reporting bias*: identified the selective reporting of results
(6) *Other bias*: identified any other sources of bias that may be present in the literature, owing to a variety of circumstances or events.

The two reviewers (VS, IJ) evaluated the level of bias within each category for each individual study by assigning a material risk of bias score (high, low, or unclear) for each of the above criteria, including supporting rationale for this score. Material bias is defined as bias significant enough to affect the results and/or conclusions of the study. Examples of criteria used to assess material bias are included in Supplementary Materials Table S3.

The support for the bias judgment is derived from the study and is highlighted by verbatim quotes from the publication, where possible. In this section, review authors may include personal comments and any relevant information supporting the rationale for their judgments. The ambiguity of information within the study can be addressed by indicating ‘probably done’ or ‘probably not done’ in addition to an explanation for why they believe so. Lastly, if the primary authors did not provide sufficient information to enable review authors to make clearly defined judgments, this should be clearly indicated.

#### 2.6.2. GRADE

The Grades of Recommendation, Assessment, Development, and Evaluation (GRADE) [40] is a systematic approach that is used to assess the quality of evidence across studies and evaluate the strength of clinical recommendations. Prior to their assessment of the quality of evidence, review authors identify the clinical outcomes on which they will be focusing. If applicable, three items must be clearly defined for each outcome, including the number of studies addressing the specific outcome of interest, the treatment comparison, and the number of participants in each comparison. Then, the quality of evidence addressing the outcomes is evaluated based on the type of evidence provided, quality points, consistency, directness, and effect size.

Two independent reviewers (VS, IJ) used the GRADE criteria to evaluate the quality of evidence across studies. The five GRADE criteria outlined by the British Medical Journal (BMJ) (2012) [40] are detailed in Supplementary Materials Table S4 and summarized below:

(1) *Type of evidence.* Scientific evidence derived from randomized control trials begins at a rating of four points; in contrast, evidence from observational studies is assigned a rating of two.
(2) *Quality points.* A total of three points can be deducted under this category based on inadequacies in follow-up procedures, sparse data, blinding, allocation concealment, and attrition.
(3) *Consistency.* Heterogeneous studies are evaluated under this category, as long as they all address the same outcomes and interventions. A quality point is deducted under this category for inconsistent results between studies while, in contrast, a quality point is added if a dose-response effect is observed or if adjustment of confounders increased the effect size.
(4) *Directness.* A maximum of two points can be deducted for issues affecting the generalizability of the results to the population of interest. Examples of issues affecting directness include co-interventions that are being tested alongside the intervention of interest, as well as the use of samples that are either too broad or too restricted.
(5) *Effect Size.* The GRADE criteria add a quality point for an odds ratio (OR) or relative risk (RR) ≥2 and adds two quality points for an OR or RR ≥5. One quality point is added for effect sizes >2 (or <0.5), while two quality points are added for effect sizes that are >5 (or <0.2) and are all statistically significant. No quality points are added for effect sizes <2 or statistically insignificant results.

When calculating the final GRADE score for each outcome, a score of at least four points indicates a high quality of evidence, three points suggests a moderate quality of evidence, two points reflects a low quality of evidence, and a score of one or less represents a very low quality of evidence. GRADE scores for independent outcomes are presented in table format, where explanations for the scores and judgments about the quality of evidence are provided. The overall interpretation of the GRADE score does not reflect the methodological quality of a single piece of literature, but rather is a measure of the quality rating of the overall evidence across studies addressing a specific outcome within the target population.

## 3. Results

### 3.1. Intervention Studies

#### 3.1.1. Study Description

The present review identified 14 intervention studies published between 1998–2018 that aimed to increase dairy and/or calcium consumption. Of the 14 intervention studies identified, seven (50%) targeted total dairy intake, six (43%) targeted total milk intake, and one (7%) targeted calcium intake; all did so as part of a larger dietary intervention. All of the intervention studies targeted children between the ages of 1.5–5 years, their parents, and/or teachers, as it was acknowledged that interventions may engage the child’s caregiver(s), but not be applied to the preschool-aged child(ren). The search methods were exhaustive and retrieved studies conducted globally, allowing for a comprehensive analysis of interventions for populations with differing baselines and habitual dairy and/or calcium intake.

#### 3.1.2. Effectiveness

Intervention effectiveness was determined as a statistically significant (*p* < 0.05) increase in dairy and/or calcium intake. Of the 14 interventions included in the review, five were effective, eight were ineffective, and one did not provide any information on the effectiveness of the intervention (Table 1). Of the five effective interventions, one targeted both parent and child, two targeted only the parent, and two targeted the child alone; importantly, both interventions targeting the child demonstrated effectiveness. The overall intervention effectiveness results reported in this review were lower than those of previously published systematic literature reviews [17,18].

One intervention [30] did not report a statistically significant change in dairy consumption from baseline to six-week post-intervention follow-up, but reported a statistically significant increase in dairy consumption using a treatment-by-time interaction model at 28 weeks post-intervention booster follow-up. (A booster in this context is a reintroduction of the intervention some time after the initial intervention has concluded; it is used to determine whether the behavior changes that were taught/implemented in the initial intervention were maintained over time).

#### 3.1.3. Sample Size, Control Groups, Effect Size

The sample size of intervention studies ranged from very small groups of parents (*n* = 7) and children (*n* = 6) as presented in the study of Kopetsky (2017) [25], to large groups of children (*n* = 3112) enrolled in the Food and Nutrition Services Food Stamp Nutrition Education Program as studied by Cason (2001) [19]. In addition, four (29%) interventions did not have a control group, while only seven (50%) interventions included in the review provided adequate information to calculate effect size. The other 50% had insufficient data (i.e., did not have a control group), or did not provide the data in the correct format to enable effect size calculation.

### 3.2. Intervention Intensity

The summary of the intervention intensity rating categories associated with effectiveness and overall intervention intensity results are presented in Table 2 and Table 3, respectively. In this review, three (23%) interventions were of low intensity, five (~38%) interventions were of medium intensity, and five (~38%) interventions were of high intensity. More than half (60%) of the medium-intensity interventions and 66.7% of the low-intensity interventions reported statistically significant (*p* < 0.05) increases in dairy and/or calcium intakes, whereas none of the high-intensity interventions were effective at increasing dairy and/or calcium consumption.

When evaluating the individual intensity rating categories, no relationships appear to exist between duration, frequency of contact or level of personalization, and intervention effectiveness (Table 2). Only one intervention had a duration of 6 to 11 weeks and demonstrated effectiveness. Additionally, the two interventions [28,32] involving contact with participants multiple times per week were effective. None of the levels of personalization were consistently linked with effectiveness, as all were used in both effective and ineffective interventions. Consistent with a previously published systematic literature review [18], all of the studies in which the intervention was conducted and applied in only one setting/environment were effective. Of the six studies using a reach of one setting, five (83.3%) demonstrated significant increases in dairy and/or calcium consumption.

To further assess intervention intensity, the interventions were divided into four groups based on the target population(s): parent and child; parent; child; or childcare services (Table 3). Child-focused interventions had a group score of 9.5 (*n* = 2), indicating a low overall intervention intensity score; however, the interventions in both studies resulted in statistically significant increases in dairy consumption. Interventions that targeted both parent and child had the highest overall group intensity score of 13.5, although only one (14.3%) of the seven interventions resulted in significantly increased calcium consumption. Overall, there was heterogeneity in terms of effectiveness across different categories of intensity and with overall intensity.

### 3.3. Behavior Change Techniques

The interventions employed a variety of behavior change techniques (BCT). Table 4 outlines the frequency of BCT associated with intervention effectiveness. Salehi et al. (2004) [31] was not included in the BCT analysis, as information about intervention effectiveness was not provided.

The most commonly used BCT, which was used in all 13 interventions, was action planning. Other commonly used BCTs were goal setting (behavior) (*n* = 12), providing information on when and where to perform the behavior (*n* = 12), providing instruction on how to perform the behavior (*n* = 12), and prompting practice (*n* = 12). There was one BCT that was used exclusively in the effective intervention by Marquis et al. (2014) [27], which was providing normative information about others’ behavior (i.e., providing information about others’ behaviors and whether they are common or uncommon in the population). Action planning and environmental restructuring were similarly used in all five effective studies, although both were also used in several ineffective studies. Action planning demonstrated 38.5% efficacy, and environmental restructuring demonstrated 62.5% efficacy. Goal setting (behavior) [19,24,27,28], generalization of the target behavior [19,24,27,28], providing information on when and where to perform the behavior [19,24,27,28], providing instruction on how to perform the behavior [19,24,27,28], teaching how to use prompts/cues [19,24,28,32], and prompt practice [19,24,27,28] were techniques used in four of the five effective interventions. Goal setting (behavior) demonstrated 33.3% efficacy, the generalization of target behavior demonstrated 36.4% efficacy, providing information on when and where to perform the behavior demonstrated 33.3% efficacy, providing instruction on how to perform the behavior had 33.3% efficacy, teaching to use prompts/cues demonstrated 57.1% efficacy, and prompting practice had 33.3% efficacy. The results demonstrate that intervention effectiveness is independent of BCT.

### 3.4. WIDER

Table 5 provides a summary of WIDER recommendations for each intervention study. Only seven of the 14 studies provided adequate descriptions of their intervention. Similarly, 57% of the studies adequately classified their change processes and design principles. Only three (21%) interventions provided access to intervention protocols, which made it difficult to further evaluate the risk of reporting bias. Four studies (29%) did not have a control group, which classified them under unclear risk of bias for random sequence generation and high risk of bias for allocation concealment. Of the 10 studies that had a control group, only three (30%) had an active control, with two of them providing adequate descriptions of the control.

### 3.5. Risk of Bias

**Figure 2:**
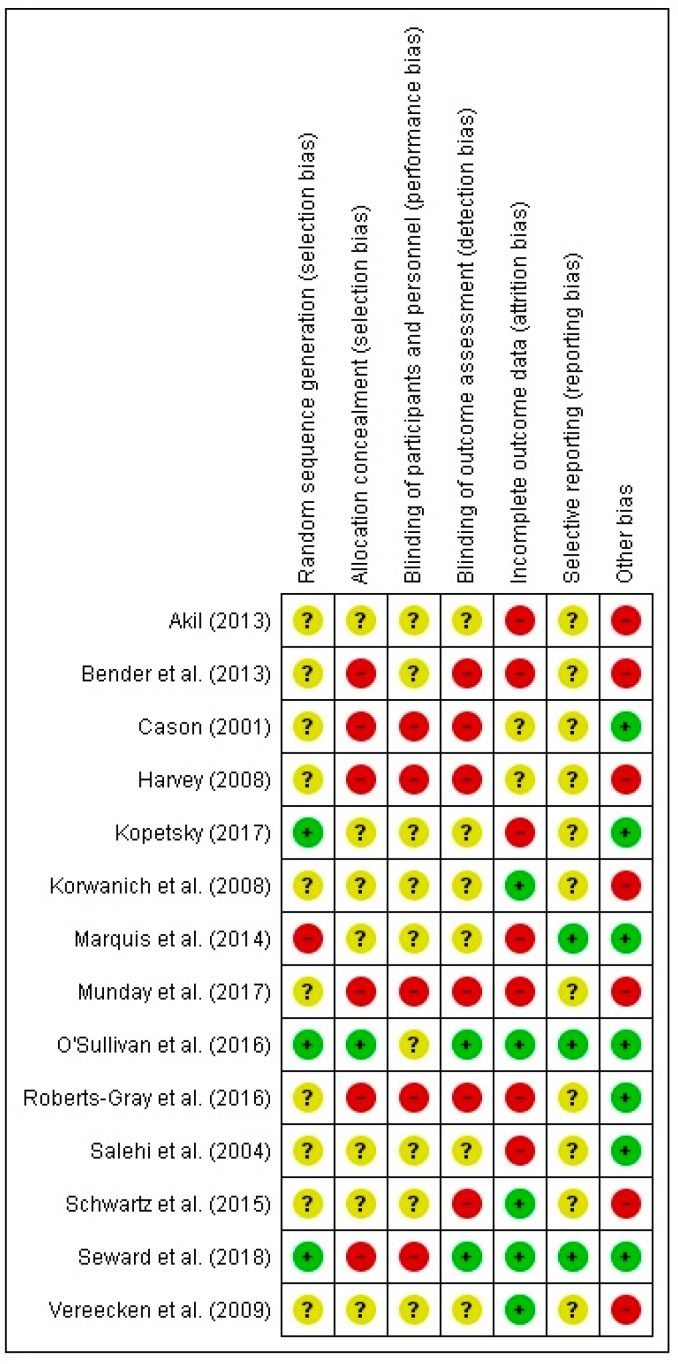
Risk of bias summary: review authors’ judgments about each risk of bias item for each included intervention study. Red, yellow, and green circles represent high, unclear, and low risk of bias, respectively.

All 14 intervention studies [19,20,22,23,24,25,26,27,28,29,30,31,32,33] included in the review were assessed for risk of bias. Figure 2 provides a summary of the authors’ (VS, IJ) judgments regarding each risk of bias item for the included intervention studies. The study with the lowest overall risk of bias was O’Sullivan et al. (2016) [29], and the study with the highest overall risk of bias was Munday et al. (2017) [28]. Three studies, two of which were dissertations, had all categories classified as either high or unclear risk of bias [22,23,24]. Overall, most of the studies had a high or unclear risk of bias in a majority of the categories.

**Figure 3:**
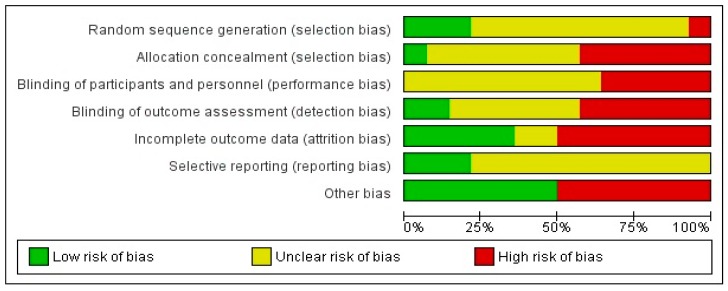
Risk of bias graph: review authors’ judgments about each risk of bias item, which are presented as percentages across all of the included intervention studies (*n* = 14).

Figure 3 presents the percentages of each risk of bias item across the 14 intervention studies. There was a high percentage (71%) of studies with an unclear risk of bias for random sequence generation. Additionally, six studies inadequately described allocation concealment. All of the studies either had a high or unclear risk of bias in the blinding of participants and personnel. Similarly, only two studies had a low risk of bias in the blinding of outcome assessment. Compared to other categories, there was a high percentage (36%) of studies that had a low risk of bias in incomplete outcome data. For the selective reporting domain, 11 studies had an unclear risk of bias, as they failed to provide protocols, and thus reviewers were unable to make clear judgments about bias risk. Half of the studies (*n* = 7) had a high risk of other bias due to self-reporting, convenience sampling, crossover bias, and underreporting. A high risk of bias was reported in 50% of the studies across the categories ‘other bias’ and ‘attrition bias’, resulting in these two categories having the highest percentage of a high risk of bias. Similarly, the highest percentage (50%) of a low risk of bias was observed in the ‘other bias’ category. Overall, in four of the seven domains, the percentage of studies with an unclear risk of bias was 50% or greater.

### 3.6. GRADE

Thirteen studies were included in the GRADE analysis. One study could not be included, as it was the only intervention assessing calcium intake as an outcome [28]. Table 6 provides a summary of GRADE results for the studies (*n* = 7) that had total dairy intake as an outcome. The overall quality of evidence across these studies was very low, as it received an overall score of zero. Table 7 provides a summary of GRADE results for the studies (*n* = 6) that had total milk intake as an outcome. Similar to total dairy intake, the overall quality of evidence across these studies was also very low, receiving an overall score of one.

The type of evidence across both outcomes received a score of +4, as all of the studies that were included in the review were intervention studies. Total dairy intake received the lowest score (−3) for quality points, as a majority of the studies had <200 participants and had a relatively high risk of bias. Conversely, total milk intake had the lowest score (−1) for consistency, as there was variability in the reporting of milk outcomes. Both outcomes received the same scores for directness (−1) and effect size (0). Overall, these results demonstrate heterogeneity between intervention studies.

## 4. Discussion

The objective of this review was to identify the characteristics of effective interventions aimed at increasing dairy and/or calcium consumption in preschool-aged children. Intervention reporting was evaluated using intervention intensity analysis, behavior change technique taxonomy, and WIDER, with risk of bias and GRADE used to assess the quality of the intervention studies. Only five (35.7%) interventions included in the review reported significant increases in dairy and/or calcium consumption post-intervention, which was lower than the ~70% reported in previously published reviews in other populations [17,18]. Characteristics associated with effectiveness included those interventions delivered in one setting (i.e., preschools, early education centers and/or daycares) versus those delivered in multiple settings, those that included the selected behavior change techniques of environmental restructuring and teach to use prompts/cues, and those that targeted both the parent and child.

The most notable finding was the lack of effectiveness reported by intervention studies aiming to increase dairy and/or calcium intakes in preschool-aged children. The ineffectiveness of most interventions may be attributed to the lack of focus on dairy or calcium intake as the targeted message. All of the interventions in the final analysis included dairy and/or calcium intakes as part of a larger dietary intervention designed to encourage healthy eating and positive dietary habits in preschool-aged children. Hendrie et al. (2012) [17] concluded that interventions specifically targeting dairy or calcium intake independent of other dietary changes were more likely to be effective than those considering dairy/calcium intake in the context of a broader message, such as general healthy eating. These findings suggest that mixed dietary interventions may dilute the impact or preclude adequate communication and/or the adaption of more targeted health and dietary messages. A possible explanation could be that less time and/or effort are allocated to dairy-specific messaging when presented as part of a mixed dietary intervention. Resource availability may also be limited when engaging in broader dietary interventions, directly impacting dairy or calcium messaging and the extent of education and communication about dairy and calcium consumption. The effectiveness of increasing dairy and calcium consumption as part of a larger dietary intervention may be influenced by the behavior change techniques implemented. For example, behavior change techniques that are effective at increasing fruit and vegetable consumption may not be similarly effective at increasing dairy and calcium intake, and therefore may impact the communication of dairy and calcium health messages.

The ineffectiveness of dairy interventions may also be attributed to the heterogeneity of the interventions. The heterogeneity observed between populations may have introduced variability in the communication of dietary messages on the basis of cultural and/or societal norms. For example, Salehi et al. (2004) [31] conducted their interventions on Qashqa’i tribe families in Iran, while Marquis et al. (2014) [27] studied a cohort of rural Ghanaian children. Salehi et al. (2004) [31] aimed to change/improve the customs and cultural practices of the Qashqa’i tribe families through educational program topics such as sanitary waste disposal, water supply, and general dietary consumption guidelines. In contrast, Marquis et al. (2014) [27] focused on improving entrepreneurial training and nutrition education to increase household access to animal food products in rural Ghana. These examples highlight the differences in the availability of resources and/or food sources between cultures, and how this could impact the extent of the dietary health and messaging provided. Secondly, heterogeneity was also observed across participants, with variability in the targets of the interventions (i.e., parent and child, parent, child, or childcare services). The variability observed across intervention targets may significantly influence dietary messaging given that interventions targeting preschool children focus more on simply increasing dairy consumption, whereas those targeting adults are more likely to emphasize the importance or relevance of doing so. Thirdly, heterogeneity existed between the approaches used to communicate dietary messaging, including mass communication, targeted communication, or tailored communication [41]. Mass communication is more generic and most likely used in environmental or group interventions, as this type of communication enables general messages to be delivered to many individuals; in contrast, tailored communication is used when the health messages are specifically designed to individual need. The ineffectiveness and heterogeneity observed across interventions make it difficult to draw reliable conclusions, highlighting the need for future intervention designs to specifically and solely target dairy and/or calcium intake in preschoolers.

The characteristics of effective interventions were determined using intervention intensity analysis. No associations were observed between intervention intensity group scores and intervention effectiveness. Moreover, interventions targeting the parent and child demonstrated the highest intervention intensity scores, but only one of the seven studies demonstrated effectiveness. Both interventions targeting the child alone, by comparison, exhibited low intervention intensity scores, but were effective at increasing dairy intakes. These observations suggest that targeting the child alone may enhance the effectiveness of interventions; although, based on the young age of the preschool children, it is unreasonable to assume that these children would be able to change their intakes without the guidance from their parents and/or caregivers. Targeting both the parent and child may demonstrate higher efficacy in increasing dairy and/or calcium intakes in preschool children, as evidence suggests that parents play an important role in developing preschoolers’ eating habits through a variety of mechanisms, including modeling of dietary behaviors [42,43], parental feeding behaviors [44,45], and the availability/accessibility of food products in the home environment [46,47]. Recent evidence from the Guelph Family Health Study, Canada, extended these findings: both mothers’ and fathers’ involvement of children in meal preparation was associated with lower child nutrition risk (mother beta = −3.45, *p* = 0.02; father beta = −1.74, *p* = 0.01) and healthy home environment scores (mother beta = −8.36, *p* < 0.001; father beta = −2.69, *p* = 0.04) [43]. These results demonstrate the strong parental influence on preschoolers’ dietary intakes, supporting the need to target parents as well as children in interventions aiming to increase dairy and/or calcium intakes. Furthermore, when considered as independent categories, no associations were evident between intervention duration, frequency of contact or level of personalization, and overall intervention effectiveness. In contrast, intervention setting did appear to be associated with overall effectiveness, as it was observed that the majority of effective interventions used a reach of only one setting. This is consistent with Marquez et al. (2015) [18], who also reported an 81.8% intervention effectiveness using a reach of one setting, suggesting that a targeted and focused intervention delivered in a single setting may be preferable to interventions delivered across multiple settings. Based on the results of this study, interventions targeting dairy and/or calcium intake should focus on delivery in preschools, early education centers, and/or daycares. Nonetheless, despite the inclusion of 14 interventions in this analysis, the validity of our conclusions regarding the relationship between intervention intensity and effectiveness is limited by the small number of studies in any given category.

Analysis of behavior change techniques was used to investigate the relationship between the use of specific techniques and intervention effectiveness. Overall, the main finding from this analysis indicates some associations between the use of specific behavior techniques and intervention effectiveness. Specifically, environmental restructuring and teaching to use prompts/cues appear to be associated with overall intervention effectiveness. These two techniques are also related, because environmental restructuring prompts the participant to alter their environment to support the target behavior (i.e., put up posters/images) and teaching to use prompt/cues teaches the individual to identify environmental cues to prompt the target behavior. Given that these behavior change techniques are strongly associated with intervention effectiveness, altering the environment to support and encourage dairy/calcium consumption may be effective in the preschool population. As further support, Marquez et al. (2015) [18] reported 66.7% intervention effectiveness with the use of environmental restructuring, which is a result that is comparable to our review. Furthermore, no significant relationships were observed between the target population (i.e., parent and child, parent alone, child alone, or childcare services), the effectiveness of the intervention, and the use of behavior change techniques. These results are consistent with those of Marquez et al. (2015) [18], who concluded that parental involvement and support was not a significant predictor of intervention effectiveness. These findings are in contrast to those published by Hendrie et al. (2012) [17], who reported that effective studies implementing behavior change techniques specifically targeted the parents and/or family, while ineffective studies targeted only the child. Overall, intervention effectiveness appears to be independent of the majority of behavior change techniques, although environmental restructuring and teaching to use prompts/cues may encourage positive behavioral change in the preschool population.

Two assessment tools, risk of bias and GRADE, were used to evaluate the overall quality of the intervention studies included in the analysis. The majority of interventions demonstrated a high or unclear risk of bias in most or all of the risk of bias categories, with the quality appraisal of total dairy and total milk intake outcomes being very low based on the GRADE criteria. This demonstrates an overall lack of reliability and validity of intervention results and conclusions, suggesting a need to further develop standards and consistency in intervention design and the reporting of outcomes related to dairy/calcium intakes. Improving the reporting of outcomes will (i) enable the identification of the outcomes that are most meaningful and relevant in the preschool population, (ii) enable the development of a consensus in this field of research regarding definitions and measures of dairy/calcium outcomes (i.e., servings per day versus amount consumed), and (iii) identify those outcomes that are most likely to promote increases in the effectiveness of dairy/calcium interventions [48]. Finally, consistency in the reporting of outcomes and clarity in intervention design would enable a comparison of study designs, sample sizes, and target populations for the purpose of determining the factors promoting the effectiveness of dairy/calcium consumption in the preschool population. Improving clarity and transparency in outcome reporting and intervention design will in turn increase the reliability and the validity of conclusions about dairy/calcium intakes in preschoolers, and inform initiatives targeting positive health behaviors.

The results of the review should be interpreted considering the limitations. One limitation was a lack of disclosure in the methodology across interventions. This made it difficult to compare the different categories of intervention intensity and determine which behavior change techniques were implemented in interventions. Studies were restricted to those published in English, which may have limited the interventions selected for final analysis. As the primary focus of this review was on interventions aiming to increase dairy and/or calcium consumption, studies that were considered ineffective in this review may have demonstrated effectiveness in changing other dietary and/or physical activity targets in the intervention. Moreover, intervention effectiveness must also be considered in context of study design, as interventions specifically designed to increase dairy and/or calcium consumption may demonstrate increased effectiveness when compared to mixed interventions. Many studies did not report the actual quantities of participants’ dairy/calcium intakes, making it difficult to report the degree of intervention efficacy. Lastly, gender bias may also be considered as a limitation, given that some studies included only one parent in the intervention, which was typically the mother. Despite these limitations, the use of several tools to both quantitatively and qualitatively review the included interventions provides a comprehensive critique of this body of literature and yields valuable insight into the characteristics of studies in this research domain.

## 5. Conclusions

Dietary interventions aiming to increase dairy and/or calcium consumption in preschool-aged children demonstrate variable success. The evidence presented in this review has identified characteristics that may enable the intervention effectiveness of increasing dairy and/or calcium intakes in preschoolers. This includes the delivery of interventions in one setting versus multiple settings, using specific behavior change techniques (such as environmental restructuring and teaching to use prompts/cues), and targeting both the parent and child. Future studies should modify interventions to exclusively target dairy and/or calcium intakes, reduce heterogeneity and/or bias, and improve transparency in the reporting of interventions. Further investigating the relationship and effect of target populations, specifically both parent and child, is another consideration, as it may be necessary and important to work with the parents and/or caregivers of these preschool children to ensure sustainable changes in dairy and/or calcium intakes. A potential avenue for future research includes exploring the use and effectiveness of booster periods for sustaining positive behavioral change over time in the preschool population. In the meantime, public health initiatives should aim to improve the dairy and calcium intake of preschool-aged children for the purpose of instilling healthy dietary habits at a young age, and mitigate the health consequences associated with insufficient intakes. Overall, the findings of this review demonstrate the need for developing effective interventions designed to increase dairy and/or calcium intakes in preschool-aged children.

## Figures and Tables

**Table 1:** Dairy and calcium intervention studies targeting preschool-aged children: study data extraction table with effect size (*d*) and intervention effectiveness.

Study	Population	Description of Intervention (I = Intervention, C = Control)	Outcome Measure (s)	Intervention Outcome Measurement (s)	Effect Size (*d*)	Effective (Y/N) ^1^
Akil (2013) [22]	Parents or caregivers of children aged 3 to 5 (*n* = 140)	I: Parents/caregivers and child followed ordinary HeadStart nutrition curriculum and participated in a nutrition education program (i.e., cooking classes, weekly nutrition newsletters)	Daily number of servings of food groups (i.e., dairy, fruits, and vegetables)	N/A. Study does not report pre/post-intervention consumption.	ND ^2^	N
		C: Parents/caregivers and children followed ordinary HeadStart nutrition curriculum				
Bender et al. (2013) [23]	Low income Hispanic mothers (18–35 years old) with children aged 3 to 5 (*n* = 33)	I: Two-phase intervention program; phase I included four biweekly interactive nutrition group lessons, and phase II included six monthly group community activities to reinforce target health behaviors (i.e., nutrition cooking classes)	Beverage (i.e., fruit juice, milk) serving size(s) and number of servings/day	Children’s baseline milk consumption in ounces per day (mean (SD)): 14.3 (0.96) Post-intervention milk consumption in ounces per day (mean (SD)): 16.8 (2.1)	ND	N
Cason (2001) [19]	Children aged 3 to 5 (male = 2990, female = 3112)	I: Children participated in a multiple intelligences theory-based nutrition education curriculum (i.e., nutrition education lessons, food tasting)	Daily number of food group servings (i.e., meat, dairy, fruit)	Reported difference in daily servings of dairy in children as mean (SD). Pre-intervention was 0.99 (1.32) and post-intervention was 2.36 (1.54).	ND	Y
Harvey (2008) [24]	Low-income African-American and Hispanic parents (*n* = 25)	I: Parents participated in a weekly nutrition education intervention; they received weekly nutrition newsletters and tracked child daily dietary servings using a kid calendar	Weekly servings for dietary components (i.e., dairy, fruits, vegetables)	Reported changes in weekly servings of low-fat dairy as mean (SD). Baseline measure was 12.44 (7.10) and week 4 post-intervention was 18.04 (7.55).	ND	Y
	Children aged 3 to 5 (female = 13, male = 12)					
Kopetsky (2017) [25]	Parent/caregiver (female = 7)	I: Parent and child attended five, 45-min nutrition education sessions on behavioral strategies (self-monitoring, parental modeling), attended education sessions on MyPlate food groups, and received weekly recipes in the mail	Dietary quality as measured by the Healthy Eating Index (HEI), 2010	HEI 2010 scores quality of dairy in the diet out of 10 points. Baseline and week 5 for dairy consumption (mean (SD)): Baseline: 8.2 (2.5) Week 5: 8.9 (2.0)	0.04	N
	Children aged 3 to 5 (male = 3, female = 3)	C: Parent and child received weekly recipes in the mail				
Korwanich et al. (2008) [26]	Parents (*n* = 219)	I: Nursery schools had implemented a newly developed healthy eating policy (i.e., advising on snack and beverage consumption at school, children engaged in nutrition education activities)	Frequency of dietary intakes per day (i.e., non-sugar milk, fresh fruit)	Frequency of non-sugar milk consumption within groups (mean (SD)). Baseline in intervention group was 0.94 (0.2) and post-intervention was 0.97 (0.2).	0.17	N
	Children aged 4 to 5 years (male = 111, female = 108)	C: No action provided in control schools				
Marquis et al. (2014) [27]	Parent/caregiver (*n* = 201)	I: Parents/caregivers attended weekly meetings for loan payments, entrepreneurship training, and nutrition education on child feeding practices	Frequency of dietary intakes per week (i.e., milk and milk products)	N/A. Study does not report pre/post-intervention consumption.	ND	Y
		C: Parents/caregivers received health education talks				
Munday et al. (2017) [28]	Parents/caregivers (*n* = 16)	I: Children participated in nutrition education sessions, food tasting sessions, sticker reward charts, kindergarten vegetable plots; parents/caregivers participated in cooking classes	Nutrient intake per day (i.e., calcium)	Reported calcium intakes as mean (SD). Baseline intake was 526 (198.4) and post-intervention was 608 (196.2).	ND	Y
	Children aged 3 to 5 (male = 13, female = 4)					
O’Sullivan et al. (2016) [29]	Mothers of children aged 3 to 5 (*n* = 149)	I: Mothers received a community-based home visiting program (i.e., provided information and instruction on parenting practice, emotional support, and access to community services), participated in the Triple P Positive Parenting Program, received child developmental materials and book packs, and were encouraged to attend healthy eating workshops	Proportion of participants meeting daily recommendations (i.e., dairy)	Intakes reported as proportion of participants in the intervention group meeting daily dairy recommendations (mean (SD)). Proportions at each of 18, 24, and 36 months were 0.74 (±0.44), 0.64 (±0.48), and 0.66 (±0.48), respectively.	1.16 to 1.94	N
	Children aged 3 to 5 (*n* = 149)	C: Mothers received child developmental materials and book packs, and were encouraged to attend healthy eating workshops				
Roberts-Gray et al. (2016) [30]	Parent–child dyads (*n* = 608)	I: Parents received nutrition newsletters and participated in parent–child activity stations; children participated in parent–child activity stations and teacher–child classroom activities; nutrition workshops implemented at the organizational level	Number of dairy servings per day	Number of dairy servings per day (mean (SD)): pre-intervention 0.73 (0.7) and post-intervention 0.79 (0.07).	0.86	N
		C: No action provided in control schools				
Salehi et al. (2004) [31]	Parents or caregivers of children <5 years of age	I: Parents/caregivers were exposed to an educational program (i.e., educated on concepts of “food pyramid”, taught daily requirements for milk and yogurt intakes)	Quantity of milk consumed (grams)	Reported quantity (g) of milk consumption at beginning of program compared to end. Beginning milk quantity (g) reported as mean (SD) was 50 (13.2), and end was 60 (9.5).	ND	N/A
	Children aged 3 to 5 (*n* = 811)	C: No action provided in control sub-tribes				
Schwartz et al. (2015) [32]	Children aged 3 to 5 (male = 40, female = 45)	I: Children were exposed to one of two feeding practices: (1) fruits, vegetables, and milk were served before the main meal (first course), and (2) fruits, vegetables, and milk were served before the main meal, and meats and grains were removed from the table after the first serving (combination)	Number of Child and Adult Care Food Program (CACFP) servings consumed per meal per day (i.e., milk)	N/A. Not reported as overall pre/post-test consumption.	−0.09 to 0.64	Y
Seward et al. (2018) [20]	Long day childcare services (*n* = 44)	I: Services were provided to staff, including training, receiving a resource pack to support the implementation of nutrition guidelines, having a dietitian complete an audit of the two-week menus, and being allocated an implementation support officer to provide advice and assistance	Number of dietary servings per day (i.e., dairy)	Reported as mean number of daily dairy servings consumed by children as mean (SD). Baseline was 0.55 (0.23) and post-intervention was 1.03 (0.57).	0.03	N
	Children aged 3 to 5 (*n* = 243)	C: Services posted a hard copy of the Caring for Children resource and received regular care from the local health district health promotion staff				
Vereecken et al. (2009) [33]	Parents (mother = 189, father = 11)	I: Children participated in guided and self-guided nutrition activities, were given feedback and reinforcement from teachers, and had access to cooking equipment and healthy foods; parents received nutrition newsletters, engaged in nutrition activities with children, and attended school activities with other parents	Average daily consumption of milk products (mL)	Reported changes in milk intakes in mL. Pre-intervention was 176 mL, and post-intervention was 153 mL. No SD reported.	−2.17	N
	Children aged 3 to 5 (male = 239, female = 237)	C: No action provided in control schools				

^1^ Intervention effectiveness is defined as a statistically significant increase (*p* < 0.05) in a dairy or calcium outcome. ^2^ Abbreviation: ND, no data or not enough data available to calculate effect size.

**Table 2:** Intervention characteristics and intensity rating categories associated with intervention effectiveness (*n* = 13).

	Effective Interventions	Ineffective Interventions	Total ^3^	% Effective ^1^
**Target of Intervention**				
Mixed	5	8	13	38.5
**Intervention Intensity**				
Low	2	1	3	66.7
Medium	3	2	5	60.0
High	0	5	5	0.0
**Duration**				
<6 weeks	2	2	4	50.0
6 to 11 weeks	1	0	1	100.0
12 weeks to 5 months	-	-	-	-
6 to 12 months	1	5	6	16.7
>12 months	1	1	2	50.0
**Frequency of Contact ^2^**				
Annually	-	-	-	-
Bimonthly to quarterly	-	-	-	-
Monthly	0	2	2	0.0
Biweekly	1	2	3	33.3
Weekly	2	5	7	28.6
Multiple times per week	2	0	2	100.0
Daily	-	-	-	-
**Level of Personalization ^2^**				
Environmental	1	1	2	50.0
Group (Parent or Child)	3	3	6	50.0
Group (Parent and Child)	-	-	-	-
Environmental + Group (Parent or Child)	1	1	2	50.0
Environmental + Group (Parent and Child)	0	2	2	0.0
Group + Individual (Parent or Child)	1	1	2	50.0
Group + Individual (Parent and Child)	0	1	1	0.0
Individual or Individual + Environmental + Group	0	2	2	0.0
**Reach**				
1 setting	5	1	6	83.3
2 settings	0	5	5	0.0
3+ settings	0	2	2	0.0

^1^ Intervention effectiveness is defined as a statistically significant increase (*p* < 0.05) in a dairy and/or calcium related outcome. ^2^ Total number of studies in Frequency of Contact and Level of Personalization will not sum to *n* = 13, because some studies used multiple frequencies of contact and multiple levels of personalization throughout the intervention. ^3^ Salehi et al. (2004) was excluded from the chart and analysis, as the authors did not provide the effectiveness of the intervention.

**Table 3:** Summary of overall intervention intensity results.

Study (*n* = 14)	Duration ^4^	Frequency ^4^	Personalization ^4^	Reach ^4^	Overall Intensity Score	Overall Intensity Rating ^2^	Effective ^3^	Group Score
**Parent and Child**								
Akil (2013) [22]	4	4	3	5	16	High	N	13.5
Bender et al. (2013) [23]	4	3.25	2.5	5	14.75	High	N	
Kopetsky (2017) [25]	1	4	4.5	3	12.5	Medium	N	
Korwanich et al. (2008) [26]	4	4 ^1^	3.5	3	14.5	High	N	
Munday et al. (2017) [28]	2	4.5	3	1	10.5	Medium	Y	
Roberts-Gray et al. (2016) [30]	1	4	3.5	3	11.5	Medium	N	
Vereecken et al. (2009) [33]	4	4 ^1^	3.5	3	14.5	High	N	
**Parent**								
Harvey (2008) [24]	1	4	2	1	8	Low	Y	12.2
Marquis et al. (2014) [27]	5	4	2	1	12	Medium	Y	
Salehi et al. (2004) [31]	4	N/A	Unclear	1	N/A	N/A	N/A	
O’Sullivan et al. (2016) [29]	5	3.5	5	3	16.5	High	N	
**Child**								
Cason (2001) [19]	4	3.5	3	1	11.5	Medium	Y	9.5
Schwartz et al. (2015) [32]	1	4.5	1	1	7.5	Low	Y	
**Childcare Services**								
Seward et al. (2018) [20]	4	3	1	1	9	Low	N	9

^1^ Frequency of contact with intervention was estimated by review authors; points of contact were divided by the overall duration of the intervention to determine an average frequency of contact. ^2^ Overall intensity rating score breakdown: low intensity (≤10.5); medium intensity (10.51 to 13.49); high intensity (≥13.5). ^3^ Intervention effectiveness is defined as a statistically significant increase (*p* < 0.05) in a dairy and/or calcium-related outcome. ^4^ Intensity ranking scale: *Duration*: 1 = ≤6 weeks; 2 = 6 to 11 weeks; 3 = 12 weeks to 5 months; 4 = 6 to 12 months; 5 = ≥12 months; *Frequency*: 1 = annually; 2 = bimonthly to quarterly; 3 = monthly; 3.5 = twice a month; 4 = weekly; 4.5 = multiple times per week; 5 = daily; *Personalization*: 1 = environmental; 2 = group (parent or child); 2.5 = group (parent and child); 3 = environmental + group (parent or child); 3.5 = environmental + group (parent and child); 4 = group with an individual component (parent or child); 4.5 = group with an individual component (parent and child); 5 = individual (parent and/or child) or individual + environmental + group (parent and/or child); *Reach*: 1 = one setting; 3 = two settings; 5 = three or more settings.

**Table 4:** Behavior change techniques associated with intervention effectiveness.

Behavior Change Technique ^1^	Effective (*N* = 5)	Ineffective (*N* = 8)	Total (*N* = 13) ^2^	% Effective ^3^
1. Provide information on consequences of behavior in general	3	5	8	37.5
2. Provide information on consequences of behavior to the individual	3	6	9	33.3
4. Provide normative information about others’ behavior	1	0	1	100.0
5. Goal setting (behavior)	4	8	12	33.3
7. Action planning	5	8	13	38.5
8. Problem solving/barrier identification	2	3	5	40.0
9. Set graded tasks	0	1	1	0.0
10. Review of behavioral goals	1	4	5	20.0
13. Rewards contingent on successful behaviors	2	2	4	50.0
15. Generalization of target behavior	4	7	11	36.4
16. Self-monitoring of behavior	1	3	4	25.0
19. Provide feedback on performance	1	6	7	14.3
20. Provide information on when and where to perform the behavior	4	8	12	33.3
21. Provide instruction on how to perform the behavior	4	8	12	33.3
22. Model/demonstrate the behavior	3	7	10	30.0
23. Teach to use prompts/cues	4	3	7	57.1
24. Environmental restructuring	5	3	8	62.5
25. Agree on behavioral contract	0	1	1	0.0
26. Prompt practice	4	8	12	33.3
27. Use of follow-up prompts	0	2	2	0.0
28. Facilitate social comparison	0	2	2	0.0
29. Plan social support/social change	2	8	10	20.0
30. Identification as a role model	2	7	9	22.2
35. Relapse prevention/coping planning	0	1	1	0.0
36. Stress management/emotional control training	0	1	1	0.0
38. Time management	0	1	1	0.0
39. General communication skills training	1	2	3	33.3

^1^ Behavior change technique numbers three, six, 11, 12, 14, 17, 18, 31, 32, 33, 34, 37, and 40 were removed from the chart and analysis as no studies employed these techniques. ^2^ Salehi et al. (2004) was excluded from the chart and analysis, as the authors did not provide the effectiveness of the intervention. ^3^ Intervention effectiveness is defined as a statistically significant increase (*p* < 0.05) in a dairy or calcium outcome.

**Table 5:** Summary of the Workgroup for Intervention Development and Evaluation Research (WIDER) recommendations.

Study (*n* = 14)	Description of Intervention	Classification of Change Process and Design Principles	Access to Intervention Manuals and/or Protocols	Description of Active Control Conditions
Akil (2013) [22]	N	N	N	N
Bender et al. (2013) [23]	Y	Y	N	No Control Group
Cason (2001) [19]	N	Y	N	No Control Group
Harvey (2008) [24]	N	N	N	No Control Group
Kopetsky (2017) [25]	Y	Y	N	Y
Korwanich et al. (2008) [26]	N	N	N	No Active Control
Marquis et al. (2014) [27]	Y	N	Y	No Active Control
Munday et al. (2017) [28]	Y	N	N	No Control Group
O’Sullivan et al. (2016) [29]	Y	Y	Y	No Active Control
Roberts-Gray et al. (2016) [30]	Y	Y	N	No Active Control
Salehi et al. (2004) [31]	N	Y	N	No Active Control
Schwartz et al. (2015) [32]	N	N	N	No Active Control
Seward et al. (2018) [20]	Y	Y	Y	Y
Vereecken et al. (2009) [33]	N	Y	N	No Active Control

**Table 6:** Summary of Grades of Recommendation, Assessment, Development and Evaluation (GRADE) results for total dairy intake outcome (*n* = 7). Intervention studies evaluated: Akil 2013 [22]; Cason 2001 [19]; Harvey 2008 [24]; Kopetsky 2017 [25]; O’Sullivan et al., 2016 [29]; Roberts-Gray et al., 2016 [30]; and Seward et al., 2018 [20].

GRADE Criteria	Rating	Support for Judgment	Overall Quality of Evidence
Type of Evidence	+4	All of the studies included were intervention studies.	High
Quality Points	−3	Multiple studies had <200 participants. The majority of studies had a high or unclear risk of bias for the blinding and allocation process, as well as attrition.	Low
Consistency	0	Most of the studies reported the ineffectiveness (*n* = 5) versus effectiveness (*n* = 2) of dairy intervention. Dairy outcomes assessed are relatively similar, as the majority of studies reported some variation of the number of servings of dairy consumed.	Moderate
Directness	−1	Generalizability of population was a limitation in several studies.	Moderate
Effect Size	0	*n* = 3 studies did not provide enough information to allow for the calculation of effect size. All of the other studies (*n* = 4) allowed for the calculation of effect size; not all of the effect sizes were >2 or <0.5 and significant.	Low
	**Overall Score:** 0		**Overall Quality of Evidence:** Very Low

**Table 7:** Summary of GRADE results for total milk intake outcome (*n* = 6). Intervention studies evaluated: Bender et al., 2013 [23]; Korwanich et al., 2008 [26]; Marquis et al., 2014 [27]; Salehi et al., 2004 [31]; Schwartz et al., 2015 [32]; and Vereecken et al., 2009 [33].

GRADE Criteria	Rating	Support for Judgment	Overall Quality of Evidence
Type of Evidence	+4	All of the studies included were intervention studies.	High
Quality Points	−1	All of the studies had a high or unclear risk of bias for blinding and allocation. Three studies had a low risk of attrition bias, with the other three studies having either a high or unclear risk of attrition bias. Generally, sparse data does not appear to be of concern, as the majority of studies had >200 participants.	Moderate
Consistency	−1	Most studies reported ineffectiveness (*n* = 3) versus effectiveness (*n* = 2) of dairy intervention, with one study not reporting effectiveness. Variability in reporting of milk outcomes; studies reported volumes of milk consumed, times consumed per day, or quantity of milk consumed in grams.	Low
Directness	−1	Generalizability of population was a limitation in multiple studies.	Moderate
Effect Size	0	*n* = 3 studies did not provide enough information to allow for the calculation of effect size. All of the other studies (*n* = 3) allowed for the calculation of effect size; not all of the effect sizes were >2 or <0.5 and significant.	Low
	**Overall Score:** 1		**Overall Quality of Evidence:** Very Low

## Supplementary Materials

The following are available at https://www.mdpi.com/2072-6643/11/4/714/s1, Search Method and Inclusion/Exclusion Criteria; Table S1: Description of the 40 behavior change techniques used in the CALO-RE Taxonomy for assessment of interventions; Table S2: Description of the four criteria of the WIDER recommendations; Table S3: Description of the Risk of Bias assessment tool; Table S4: The GRADE scoring system used to evaluate the quality of scientific evidence.
