# Supplementary material for: Interventions Aimed at Increasing Dairy and/or Calcium Consumption of Preschool-Aged Children: A Systematic Literature Review
Source: Nutrients. 2019 Mar 27;11(4):714. doi: 10.3390/nu11040714 (PMC6521222; doi:10.3390/nu11040714)
Supplement: Supplementary file 1 [file nutrients-11-00714-s001.pdf]

Supplementary Materials: Interventions Aimed at Increasing Dairy and/or Calcium Consumption of Preschool-Aged Children

The systematic literature search was conducted on 14 June 2018. All of the searches were limited by date (1998–2018) and language (English only).

Databases

1. ProQuest – includes 40 databases, including PubMed, dissertations and theses, Agricola (from the United States Department of Agriculture), ERIC (educational database), and agriculture journals
2. Web of Science (also known as Web of Knowledge) – includes PubMed, Food Science and Technology Abstracts, and conference proceedings
3. Cochrane Database – database of systematic reviews
4. CINAHL (Cumulative Index to Nursing and Allied Health Literature)
5. CAB Direct
6. PsycINFO

Search Terms

1. ProQuest

Searched ‘Document Title’ and ‘Abstract’ for all terms. Clicked ‘Remove Duplicates’ prior to searching.

INTERVENTION: “clinical trial” or “latin square” or “time series” or ((before and after) or (study or studies or trial* or design*)) or ((single* or doubl* or trebl* or triple) and (blind* or mask)) or placebo* or random* or (“matched communities” or “matched schools” or “matched populations”) or control* or (“comparison group*” or “control group*”) or “matched pairs” or (“outcome study” or “outcome studies”) or (“quasiexperimental” or “quasi experimental” or “pseudo experimental”) or (nonrandomi* or “non randomi*” or “pseudo randomi*” or “quasi randomi*”) or prospective* or volunteer* or experimental or intervention* or model or models or “intervention stud*” or “comparative study” or “evaluation study”

NUTRITION: diet* or “healthy eating” or “meal pattern*” or meal* or “sugar* beverage*” or nutr* or food or “food habits” or “feeding behavior” or “beverage*”

POPULATION: (Preschool* or child* or pre-k* or toddler* or school* or “preschool-aged children” or “young people” or “young person” or pediatr* or paediatr* or boy* or girl* or “nursery school” or “day care” or “day care centre” or “early childhood” or pre-school* or “pre-school aged children” or childcare or “childcare centre” or “child development centre”) AND (famil* or teach* or educat* or instruct* or schoolteach* or parent* or mother* or father* or paternal or maternal)

DAIRY/CALCIUM: Yogurt or dairy or milk or cheese or yoghurt or calcium or "cow’s milk" or "calcium-rich foods" or "dairy products" or "dairy foods" or "dietary calcium" or "milk intake"

2. Web of Science

Searched ‘All Document Types’ for all terms.

INTERVENTION: TS=(“clinical trial” or “latin square” or “time series” or ((before and after) or (study or studies or trial* or design*)) or ((single* or doubl* or trebl* or triple) and (blind* or mask)) or placeb* or random* or (“matched communities” or “matched schools” or “matched populations”) or control* or (“comparison group*” or “control group*”) or “matched pairs” or (“outcome study” or “outcome studies”) or (“quasiexperimental” or “quasi experimental” or “pseudo experimental”) or (nonrandomi* or “non randomi*” or “pseudo randomi*” or “quasi randomi*”) or prospective* or volunteer* or experimental or intervention* or model or models or “intervention stud*” OR “comparative study” or “Research Design” OR “Controlled Clinical Trials” OR “Randomized Controlled Trials” OR “Random Allocation” or “Double-Blind Method” OR “Single-Blind Method” OR placebos or “Evaluation Study” OR “Follow-Up Studies” OR “Prospective Studies” OR “Cross-Over Studies”)

NUTRITION: TS=(diet* or “healthy eating” or “meal pattern*” or meal* or “sugar* beverage*” or nutr* or “food habits” or “feeding behavior” or food or beverage*)

POPULATION: TS=(Preschool* or child* or pre-k* or toddler* or school* or “preschool-aged children” or “young people” or “young person” or pediatr* or paediatr* or boy* or girl* or “nursery school” or “day care” or “day care centre” or “early childhood” or pre-school* or “pre-school aged children” or childcare or “childcare centre” or “child development centre”)

TS=(famil* or teach* or educat* or instruct* or schoolteach* or parent* or mother* or father* or paternal or maternal)

DAIRY/CALCIUM: TS=(Yogurt or dairy or milk or cheese or yoghurt or calcium or "cow’s milk" or "calcium-rich foods" or "dairy products" or "dairy foods" or "dietary calcium" or "milk intake”)

3. Cochrane Database

Searched all Medical Subject Headings (MeSH) terms without exploding trees and added to search manager. Searched all keywords using ‘Title, Abstract, Keywords’.

INTERVENTION: ‘Research Design’ [Mesh] OR ‘Controlled Clinical Trials as Topic’ [Mesh] OR ‘Controlled Clinical Trial’ [Publication Type] OR ‘Randomized Controlled Trials as Topic’ [Mesh] OR ‘Random Allocation’ [Mesh] OR ‘Double-Blind Method’ [Mesh] OR ‘Single-Blind Method’ [Mesh] OR ‘Placebos’ [Mesh] OR ‘Evaluation Studies’ [Publication Type] OR ‘Evaluation Studies as Topic’ [Mesh] OR ‘Comparative Study’ [Publication Type] OR ‘Follow-Up Studies’ [Mesh] OR ‘Prospective Studies’ [Mesh] OR ‘Cross-Over Studies’ [Mesh]

“clinical trial” or “latin square” or “time series” or ((before and after) or (study or studies or trial* or design*)) or ((single* or doubl* or trebl* or triple) and (blind* or mask)) or placeb* or random* or (“matched communities” or “matched schools” or “matched populations”) or control* or (“comparison group*” or “control group*”) or “matched pairs” or (“outcome study”

or “outcome studies”) or (“quasiexperimental” or “quasi experimental” or “pseudo experimental”) or (nonrandomi* or “non randomi*” or “pseudo randomi*” or “quasi randomi*”) or prospective* or volunteer* or experimental or intervention* or model or models or “intervention stud*” OR “comparative study” or “evaluation study”

NUTRITION: ‘Diet’ [Mesh] OR ‘Food’ [Mesh] OR ‘Food Habits’ [Mesh] OR ‘Feeding Behavior’ [Mesh] OR ‘Beverages’ [Mesh].

diet* or “healthy eating” or “meal pattern*” or meal* or “sugar* beverage*” or nutr* or food or “food habits” or “feeding behavior” or “beverage*”

POPULATION: 'Child, Preschool' [Mesh] OR ‘Family’ [Mesh] OR ‘Parents’ [Mesh] OR ‘Mothers’ [Mesh] OR ‘Fathers’ [Mesh]

(Preschool* or child* or pre-k* or toddler* or school* or “preschool-aged children” or “young people” or “young person” or pediatr* or paediatr* or boy* or girl* or “nursery school” or “day care” or “day care centre” or “early childhood” or pre-school* or “pre-school aged children” or childcare or “childcare centre” or “child development centre”) AND (famil* or teach* or educat* or instruct* or schoolteach* or parent* or mother* or father* or paternal or maternal)

DAIRY/CALCIUM: 'Milk' [Mesh] OR 'Cheese' [Mesh] OR 'Yogurt' [Mesh]

Yogurt or dairy or milk or cheese or yoghurt or calcium or "cow’s milk" or "calcium-rich foods" or "dairy products" or "dairy foods" or "dietary calcium" or "milk intake”

4. <u>CINAHL</u>

Searched CINAHL Headings for MeSH terms with Major Concept. Searched ‘Title’, ‘Abstract’, and ‘Subject’ for all keywords.

INTERVENTION: ‘Study Design’ [Mesh] OR ‘Randomized Controlled Trials’ [Mesh] OR ‘Controlled Clinical Trial’ [Publication Type] OR ‘Clinical Trials’ [Mesh] OR ‘Random Assignment’ [Mesh] OR ‘Double-Blind Studies’ [Mesh] OR ‘Single-Blind Studies’ [Mesh] OR ‘Placebos’ [Mesh] OR ‘Evaluation Studies’ [Publication Type] OR ‘Evaluation Research’ [Mesh] OR ‘Comparative Study’ [Publication Type] OR ‘Prospective Studies’ [Mesh] OR ‘Crossover Design’ [Mesh]

“clinical trial” or “latin square” or “time series” or ((before and after) or (study or studies or trial* or design*)) or ((single* or doubl* or trebl* or triple) and (blind* or mask)) or placeb* or random* or (“matched communities” or “matched schools” or “matched populations”) or control* or (“comparison group*” or “control group*”) or “matched pairs” or (“outcome study” or “outcome studies”) or (“quasiexperimental” or “quasi experimental” or “pseudo experimental”) or (nonrandomi* or “non randomi*” or “pseudo randomi*” or “quasi randomi*”) or prospective* or volunteer* or experimental or intervention* or model or models or “intervention stud*” OR “comparative study” or “evaluation study”

NUTRITION: ‘Diet’ [Mesh] OR ‘Food’ [Mesh] OR ‘Food Habits’ [Mesh] OR ‘Beverages’ [Mesh].

diet* or “healthy eating” or “meal pattern*” or meal* or “sugar* beverage*” or nutr* or food or “food habits” or “feeding behavior” or “beverage*”

POPULATION: 'Child, Preschool' [Mesh] or Preschool* or child* or pre-k* or toddler* or school* or “preschool-aged children” or “young people” or “young person” or pediatr* or paediatr* or boy* or girl* or “nursery school” or “day care” or “day care centre” or “early childhood” or pre-school* or “pre-school aged children” or childcare or “childcare centre” or “child development centre”

‘Family’ [Mesh] OR ‘Parents’ [Mesh] OR ‘Mothers’ [Mesh] OR ‘Fathers’ [Mesh] OR famil* or teach* or educat* or instruct* or schoolteach* or parent* or mother* or father* or paternal or maternal

DAIRY/CALCIUM: 'Milk' [Mesh] OR 'Cheese' [Mesh] OR 'Yogurt' [Mesh]

Yogurt or dairy or milk or cheese or yoghurt or calcium or "cow’s milk" or "calcium-rich foods" or "dairy products" or "dairy foods" or "dietary calcium" or "milk intake”

5. <u>CAB Direct</u>

Searched ‘Article Title’, ‘Abstract’, and ‘Subject Term’ for all terms.

INTERVENTION: “clinical trial” or “latin square” or “time series” or ((before and after) or (study or studies or trial* or design*)) or ((single* or doubl* or trebl* or triple) and (blind* or mask)) or placeb* or random* or (“matched communities” or “matched schools” or “matched populations”) or control* or (“comparison group*” or “control group*”) or “matched pairs” or (“outcome study” or “outcome studies”) or (“quasiexperimental” or “quasi experimental” or “pseudo experimental”) or (nonrandomi* or “non randomi*” or “pseudo randomi*” or “quasi randomi*”) or prospective* or volunteer* or experimental or intervention* or model or models or “intervention stud*” or “comparative study” or “research design” or “controlled clinical trials” or “randomized controlled trials” or “random allocation” or “double-blind method” or “single-blind method” or placebos or “evaluation study” or “follow-up studies” or “prospective studies” or “cross-over studies”

NUTRITION: diet* or “healthy eating” or “meal pattern*” or meal* or “sugar* beverage*” or nutr* or “food habits” or “feeding behavior” or food or beverage*

POPULATION: (Preschool* or child* or pre-k* or toddler* or school* or “preschool-aged children” or “young people” or “young person” or pediatr* or paediatr* or boy* or girl* or “nursery school” or “day care” or “day care centre” or “early childhood” or pre-school* or “pre-school aged children” or childcare or “childcare centre” or “child development centre”) AND (famil* or teach* or educat* or instruct* or schoolteach* or parent* or mother* or father* or paternal or maternal)

DAIRY/CALCIUM: Yogurt or dairy or milk or cheese or yoghurt or calcium or "cow’s milk" or "calcium-rich foods" or "dairy products" or "dairy foods" or "dietary calcium" or "milk intake”

6. PsycINFO

Searched all keywords in 'Title', 'Abstract', and 'Keywords'.

INTERVENTION: "clinical trial" or "latin square" or "time series" or ((before and after) or (study or studies or trial* or design*)) or ((single* or doubl* or trebl* or triple) and (blind* or mask)) or placeb* or random* or ("matched communities" or "matched schools" or "matched populations") or control* or ("comparison group*" or "control group*") or "matched pairs" or ("outcome study" or "outcome studies") or ("quasiexperimental" or "quasi experimental" or "pseudo experimental") or (nonrandomi* or "non randomi*" or "pseudo randomi*" or "quasi randomi*") or prospective* or volunteer* or experimental or intervention* or model or models or "intervention stud*" or "comparative study" or "research design" or "controlled clinical trials" or "randomized controlled trials" or "random allocation" or "double-blind method" or "single-blind method" or placebos or "evaluation study" or "follow-up studies" or "prospective studies" or "cross-over studies"

NUTRITION: diet* or "healthy eating" or "meal pattern*" or meal* or "sugar* beverage*" or nutr* or "food habits" or "feeding behavior" or food or beverage*

POPULATION: (Preschool* or child* or pre-k* or toddler* or school* or "preschool-aged children" or "young people" or "young person" or pediatr* or paediatr* or boy* or girl* or "nursery school" or "day care" or "day care centre" or "early childhood" or pre-school* or "pre-school aged children" or childcare or "childcare centre" or "child development centre") AND (famil* or teach* or educat* or instruct* or schoolteach* or parent* or mother* or father* or paternal or maternal)

DAIRY/CALCIUM: Yogurt or dairy or milk or cheese or yoghurt or calcium or "cow's milk" or "calcium-rich foods" or "dairy products" or "dairy foods" or "dietary calcium" or "milk intake"

Inclusion Criteria

1) Intervention studies, with or without a control group
2) Interventions to modify dietary intake (must include dairy/calcium intake as a measurement)
3) Interventions aimed at increasing dairy consumption in preschool-aged children (1.5 to 5 years old), including any studies that target families, schools, and/or early education centres
4) Outcomes included dairy and/or calcium intake; reported at group level, individual level, or both

Exclusion Criteria

1) Clinical populations (ie. obesity and lactose intolerance)
2) Studies including calcium supplementation
3) Publications older than 20 years (i.e., published prior to 1998)
4) Non-English publications
5) Case studies

Supplementary Materials: Tables

Table S1: Description of the 40 behavior change techniques used in the Coventry, Aberdeen, and London-Refine (CALO-RE) taxonomy for assessment of interventions. Information retrieved from Michie et al. (2011).

Behavior Change Technique	Description
1. Information on consequences of behavior in general	Provide information about the behavior and the consequences in the general case; most often based on epidemiological data.
2. Information on consequences of behavior to the individual	Provide information about the costs and benefits of performing or not performing the behavior(s) to the participants and/or the group of participants. The information provided can include costs and/or benefits, and do not have to be health-related (i.e., feelings).
3. Information about others' approval	This involves determining what other people think of the participants behavior(s). It identifies whether people approve or disapprove of what the participant is doing or is going to do.
4. Provide normative information about others' behavior	This provides information about others' behaviors and whether they are common or uncommon in the population.
5. Goal setting (behavior)	The participant is aided in making a behavioral change by encouraging them to change and/or maintain change.
6. Goal setting (outcome)	Participant is encouraged to set a goal that can be achieved through a specified behavior, but is not defined by the behavior (i.e., lowering blood pressure).
7. Action planning	Detailed planning of what the participant will do, including when, where, and in what situation.
8. Problem solving/barrier identification	Participant developing a plan to change their behavior(s). The participant is required to think about potential barriers and solutions to overcome them. Examples of barriers include social, cognitive, emotional, or environmental barriers.
9. Set graded tasks	Participant breaking down the target behavior into smaller and more manageable tasks, allowing for the participant to build up to performing the target behavior.
10. Review of behavior goals	Reviewing and/or analyzing previous behavior goals that were set to ensure they were achieved. Oftentimes, this requires setting goals, a participant acting on the goals, and then a follow-up to readjust and/or reconsider any goals set.
11. Review of outcome goals	Review of the previous outcome goals set (i.e., to lower blood pressure). This involves previously setting an outcome goal, participant attempting to achieve the set outcome goal, and then revising any goals as required.
12. Rewards contingent on behavior or progress toward a behavior	Praising and/or rewarding the participant for attempting to achieve a set behavioral goal. This includes any effort and/or

	progress that is made by the participant to perform the target behavior.
13. Rewards contingent on successful behaviors	Reinforcing the successful performance of the target behavior(s). This includes encouragement, praise, and/or material rewards, although the reward must be linked to successful completion of the target behavior.
14. Shaping	Involves a graded use of contingent rewards. Rewards are first given to a participant for achieving any approximation of the target behavior (i.e., increasing physical activity). Rewards are then given to participants for completing a more demanding performance of the target behavior (i.e., moderate physical activity for 30 minutes 3 times per week).
15. Generalization of target behavior	The participant is encouraged to perform the target behavior in varying situations to ensure that the behavior is not isolated to a particular condition, but rather integrated into a variety of contexts within the participant's life.
16. Self-monitoring of behavior	The participant is asked to maintain a record of the target behavior to inspire changing behavior. Examples include keeping a diary or completing a questionnaire.
17. Self-monitoring of behavioral outcomes	Participants maintain records of measurements that may have influence over their target behavior (i.e., weight loss, blood pressure).
18. Focus on past success	Participants contemplate/reflect on previous examples of successful target behavior in their lives.
19. Provide feedback on performance	Experts can offer feedback to participants using their recorded behavior data or comment on their overall behavioral performance.
20. Provide information on where and when to perform the behavior	Experts inform participants (verbally or written) as to when and where the target behavior should be performed.
21. Provide instruction on how to perform the behavior	Requires that experts instruct participants on how to perform the target behavior, either written or verbally.
22. Model/demonstrate the behavior	Experts show the participants how to perform the target behavior, either through visual or physical demonstrations that occur in person or remotely.
23. Teach to use prompts/cues	Participants are taught how to recognize environmental cues that remind and/or prompt them to perform the target behavior. Examples of cues include time of day or cell phone alerts.
24. Environmental restructuring	Participants are encouraged to modify their environment to ensure it supports their target behavior. For example, those who want to lose weight may be asked to throw away high-calorie snacks.
25. Agree behavioral contract	Participants sign a written contract outlining the target behavior so that there is a written record of each participant's resolution.

26. Prompt practice	Interveners should encourage the participants to practice and repeat the target behaviors multiple times.
27. Use of follow-up prompts	The components of the intervention are reduced in duration, frequency, and intensity over time.
28. Facilitate social comparison	Encouraging participants to compare their behavior performance to their peers.
29. Plan social support/social change	Participants should plan strategies to gain social support from their surrounding network (friends, family) in order to enable them to perform and achieve their target behavior/outcomes (i.e., buddy system).
30. Identification as a role model	Participants should focus on how they can be an example to others and positively influence their behavior.
31. Prompt anticipated regret	Interveners should induce feelings of regret in participants about performing or not performing the target behavior. This will allow participants to explore their feelings should they not perform the specified behavior.
32. Fear arousal	Involves informing participants of risk and/or mortality information related to certain behaviors, using emotive images as a means of evoking a fearful response.
33. Self-talk	Encourage participants to talk to themselves (silently or aloud) before and during the behaviors to maintain action.
34. Use of imagery	Educate the person on how to successfully visualize behavior performance.
35. Relapse prevention/coping planning	Planning how to maintain a behavior that has been changed. The participant is asked to identify situations in which the changed behavior may not have been adequately maintained, and then develop a strategy to manage and/or avoid those situations in the future.
36. Stress management/emotional control training	This is a set of techniques that aims to reduce stress and anxiety in participants in order to facilitate performance of the target behavior.
37. Motivational interviewing	A clinical method using techniques to engage in change talk to help minimize the participant resisting behavior change.
38. Time management	Includes techniques that teach participants how to manage their time to enable target behavior performance.
39. General communication skills training	This includes techniques that aim to improve the general communication skills of participants. This can include participating in group events focused on listening skills or role play.
40. Stimulate anticipation of future rewards	Interveners create the anticipation of future rewards without actively rewarding the participants' behavior throughout the intervention period. For example, this technique is coded when participants are told at the onset that they will be rewarded based on achievement of the target behavior.

Table S2: Description of the four criteria of the WIDER recommendations, and associated supplementary recommendations, used for evaluating the reporting of the components of behavior change interventions. Information retrieved from Albrecht et al. (2013).

WIDER Recommendations	Supplementary Recommendations
Description of interventions in published papers	1. Characteristics of those delivering the intervention<br>2. Characteristics of the recipients<br>3. The setting<br>4. The mode of delivery<br>5. The intensity<br>6. The duration<br>7. Adherence to delivery protocols<br>8. Detailed description of the intervention content provided for each study group
Classification of change process and design principles	1. The intervention development<br>2. The change techniques used in the intervention<br>3. The causal processes targeted by the change techniques
Access to intervention manuals and/or protocols	Reviewers submitted protocols or manuals for publication to make the supplementary materials easily accessible (i.e., online)
Description of active control conditions	1. Characteristics of those delivering the control<br>2. Characteristics of the recipients<br>3. The setting<br>4. The mode of delivery<br>5. The intensity<br>6. The duration<br>7. Adherence to delivery protocols<br>8. Detailed description of the control content provided

Table S3: The Cochrane Collaboration criteria used to judge the risk of bias in the "Risk of Bias" assessment tool. Information retrieved from Higgins and Green (2011).

**Selection Bias**		
Random Sequence Generation	Criteria for judgment of "low risk" of bias	Description of a random component in the sequence generation process such as:<br>• Random number table<br>• Use of a computer random number generator<br>• Tossing a coin<br>• Shuffling cards or envelopes<br>• Throwing dice<br>• Minimization
	Criteria for judgment of "high risk" of bias	Description of a non-random component in the sequence generation process such as:<br>• Sequence generated by even or odd date of birth<br>• Sequence generated by rule based on clinical or hospital record number<br>• Allocation by clinician judgment<br>• Allocation based on preference of participant
	Criteria for judgment of "unclear risk" of bias	Information provided about the sequence generation process was not sufficient to allow for judgment of "low risk" or "high risk" of bias
Allocation Concealment	Criteria for judgment of "low risk" of bias	Investigators and participants could not predict assignment because one of the following methods was used to conceal allocation:<br>• Central allocation (web-based, telephone)<br>• Sequentially numbered drug containers of identical appearance<br>• Opaque, sequentially numbered, sealed envelopes
	Criteria for judgment of "high risk" of bias	Investigators and participants could possibly predict assignment because allocation was based on one of the following:<br>• Used an open random allocation schedule<br>• Envelopes were assigned without proper safeguards (i.e., were unsealed)<br>• Date of birth<br>• Any other unconcealed procedure
	Criteria for judgment of "unclear risk" of bias	Information provided about the allocation concealment process was not described or was not sufficiently described to allow for judgment of "low risk" or "high risk" of bias
**Performance Bias**		
Blinding of Participants and Personnel	Criteria for judgment of "low risk" of bias	Any of the following:<br>• No blinding or lack of blinding, but review authors judge that the outcome is not likely to be affected by lack of blinding<br>• Blinding of participants and personnel is ensured
	Criteria for judgment of "high risk" of bias	Any of the following:

<table>
<tr><td></td><td></td><td>No blinding or incomplete blinding; review authors judge that outcome is likely to be affected by lack of blinding
Blinding of participants and personnel is attempted, but blinding could have been broken, and outcome is likely to be affected by lack of blinding</td></tr>
<tr><td></td><td>Criteria for judgment of “unclear risk” of bias</td><td>Information about the blinding of participants and personnel was not discussed or was not sufficiently described to allow for the judgment of “low risk” or “high risk” of bias</td></tr>
<tr><td colspan="3">Detection Bias</td></tr>
<tr><td rowspan="3">Blinding of Outcome Assessment</td><td>Criteria for judgment of “low risk” of bias</td><td>Any of the following:
No blinding or lack of blinding, but review authors judge that the outcome measurement is not likely to be affected by lack of blinding
Blinding of outcome measure is ensured</td></tr>
<tr><td>Criteria for judgment of “high risk” of bias</td><td>Any of the following:
No blinding or incomplete blinding; review authors judge that outcome measurement is likely to be affected by lack of blinding
Blinding of outcome assessment, but blinding could have been broken, and outcome measurement is likely to be affected by lack of blinding</td></tr>
<tr><td>Criteria for judgment of “unclear risk” of bias</td><td>Information about the blinding of outcome assessments was not discussed or was not sufficiently described to allow for judgment of “low risk” or “high risk” of bias</td></tr>
<tr><td colspan="3">Attrition Bias</td></tr>
<tr><td rowspan="2">Incomplete Outcome Data</td><td>Criteria for judgment of “low risk” of bias</td><td>Any of the following:
No missing outcome data
Reasons for missing outcomes unlikely to be related to true outcome
Missing outcome data balanced across intervention groups, with similar reasons for missing outcome data across groups
Dichotomous outcome data; proportion of missing data compared with observed event risk is not enough to have a clinically significant impact on intervention effect estimate
Continuous outcome data; plausible effect size among missing outcomes not enough to have a clinically relevant impact on observed effect size
Missing data accounted for using appropriate methods</td></tr>
<tr><td>Criteria for judgment of “high risk” of bias</td><td>Any of the following:
Reason for missing outcome data is likely to be related to true outcome; could be imbalance in numbers or missing data across intervention groups
Dichotomous outcome data; proportion of missing outcomes compared with observed event risk is enough to generate a clinically relevant bias in intervention effect estimate</td></tr>
</table>

		• Continuous outcome data; plausible effect size among missing outcomes is enough to generate a clinically relevant bias in observed effect size
	Criteria for judgment of “unclear risk” of bias	Information about outcome data was not discussed or was not sufficiently described to allow for judgment of “low risk” or “high risk” of bias
**Reporting Bias**		
Selective Reporting	Criteria for judgment of “low risk” of bias	Any of the following:<br>• Study protocol is available, and all of the study’s pre-specified outcomes that were of interest in the review<br>• Study protocol is not available, but the published reports include all of the expected outcomes, including those that were pre-specified
	Criteria for judgment of “high risk” of bias	Any of the following:<br>• Not all the study’s pre-specified primary outcomes have been reported<br>• One or more of the reported primary outcomes were not pre-specified<br>• One or more outcomes in the review are reported incompletely such that they cannot be entered in a meta-analysis<br>• Study report does not include results for a key outcome that would have been expected to have been reported for such a study
	Criteria for judgment of “unclear risk” of bias	Insufficient information provided to allow for judgment of “low risk” or “high risk” of bias. It is likely that many studies will fall into this category.
**Other Bias**		
Other Bias	Criteria for judgment of “low risk” of bias	The study appears to be free of any other sources of bias.
	Criteria for judgment of “high risk” of bias	There is at least one other source of bias present. For example, the study:<br>• Had bias related to the study design<br>• Has been claimed to be fraudulent<br>• Had any other problem(s)
	Criteria for judgment of “unclear risk” of bias	There may be a risk of bias, but the study either had insufficient information to evaluate whether an important risk of bias exists or had insufficient evidence that the identified problem will introduce bias.

Table S4: The GRADE scoring system used by BMJ Clinical Evidence to evaluate the quality of scientific evidence. Information retrieved from BMJ (2012).

**Type of Evidence**		
Initial score based on type of evidence	+4	Randomized control trials or systematic reviews of randomized control trials, +/- other types of evidence
	+2	Observational evidence (i.e., case control, cohort)
**Quality**		
Based on	Blinding and allocation process	
	Follow-up and withdrawals	
	Sparse data	
	Other methodological concerns (i.e., subjective outcomes)	
Score	0	No problems
	–1	Problem with one element
	–2	Problem with two elements
	–3	Problem with three or more elements
**Consistency**		
Based on	Degree of consistency of effect between or within studies	
Score	+1	Evidence of dose response across or within studies; additional one point added if adjustment for confounders would have increased the effect size
	0	All/most studies show similar results
	–1	Lack of agreement between studies (i.e., heterogeneity in statistics between randomized control trials)
**Directness**		
Based on	Generalizability of population and outcomes from each study to population of interest	
Score	0	Population and outcomes broadly generalizable
	–1	Problem with one element
	–2	Problem with two or more elements
**Effect Size**		
Based on	The reported odds ratio/relative ratio/hazard ratio for comparison	
Score	0	Not all effect sizes >2 or <0.5 and significant; or if odds ratio/relative ratio/hazard ratio not significant
	+1	Effect size >2 or <0.5 for all studies/meta-analyses included in comparison and is significant
	+2	Effect size >5 or <0.2 for all studies/meta-analyses included in comparison and is significant
